# **Prediction of amyloid** β **PET positivity using machine learning in patients with suspected cerebral amyloid angiopathy markers**

**DOI:** 10.1038/s41598-020-75664-8

**Published:** 2020-11-02

**Authors:** Young Hee Jung, Hyejoo Lee, Hee Jin Kim, Duk L. Na, Hyun Jeong Han, Hyemin Jang, Sang Won Seo

**Affiliations:** 1 Department of Neurology, College of Medicine, Myoungji Hospital, Hanyang University, Goyang, Republic of Korea; 2 Department of Neurology, Sungkyunkwan University of School of Medicine, Samsung Medical Center, 81 Irwon-ro, Gangnam-gu, Seoul, 06351 Republic of Korea; 3 Neuroscience Center, Samsung Medical Center, Seoul, Republic of Korea; 4 Samsung Alzheimer Research Center, Research Institute for Future Medicine, Samsung Medical Center, 81 Irwon-ro, Gangnam-gu, Seoul, 06351 Republic of Korea; 5 Department of Intelligent Precision Healthcare Convergence, Sungkyunkwan University, Suwon, Republic of Korea; 6 Department of Health Science and Technology, SAIHST, Sungkyunkwan University, Seoul, Republic of Korea; 7 Stem Cell and Regenerative Medicine Institute, Samsung Medical Center, Seoul, Republic of Korea

**Keywords:** Medical research, Neurology, Risk factors

## Abstract

Amyloid-β(Aβ) PET positivity in patients with suspected cerebral amyloid angiopathy (CAA) MRI markers is predictive of a worse cognitive trajectory, and it provides insights into the underlying vascular pathology (CAA vs. hypertensive angiopathy) to facilitate prognostic prediction and appropriate treatment decisions. In this study, we applied two interpretable machine learning algorithms, gradient boosting machine (GBM) and random forest (RF), to predict Aβ PET positivity in patients with CAA MRI markers. In the GBM algorithm, the number of lobar cerebral microbleeds (CMBs), deep CMBs, lacunes, CMBs in dentate nuclei, and age were ranked as the most influential to predict Aβ positivity. In the RF algorithm, the absence of diabetes was additionally chosen. Cut-off values of the above variables predictive of Aβ positivity were as follows: (1) the number of lobar CMBs > 16.4(GBM)/14.3(RF), (2) no deep CMBs(GBM/RF), (3) the number of lacunes > 7.4(GBM/RF), (4) age > 74.3(GBM)/64(RF), (5) no CMBs in dentate nucleus(GBM/RF). The classification performances based on the area under the receiver operating characteristic curve were 0.83 in GBM and 0.80 in RF. Our study demonstrates the utility of interpretable machine learning in the clinical setting by quantifying the relative importance and cutoff values of predictive variables for Aβ positivity in patients with suspected CAA markers.

## Introduction

Cerebral amyloid angiopathy (CAA) is a cerebral small vessel disease (CSVD) characterized by amyloid β (Aβ) deposition in leptomeningeal and cortical vessels^1,2^. According to the modified Boston criteria, patients with multiple strictly lobar intracranial hemorrhage (ICH)/cerebral microbleeds (CMBs) or cortical superficial siderosis (cSS) on brain magnetic resonance imaging (MRI) are specific for CAA pathology, which leads to a clinico-radiological diagnosis of probable CAA^3,4^.

Recently, the clinical utility of Aβ proton emission tomography (PET) in CAA patients has been widely investigated^5–7^. Based on previous evidence, Aβ + PET scans in patients with CAA MRI markers may have clinical utility in two ways. First, Aβ positivity in CAA patients enables clinicians to predict the prognosis of cognitive trajectories. Our previous study showed that Aβ + patients with probable CAA had worse cognitive trajectories than their Aβ- counterparts^7^. Several studies have emphasized the clinical significance of Aβ + PET scans in assessing cognition in neurodegenerative diseases, including MCI^8^, AD, and vascular cognitive impairment^9–11^. Second, Aβ PET positivity may provide insights into the underlying vascular pathology in patients with suspected CAA MRI markers; clinicians encounter patients with several lobar CMBs combined with a few deep CMBs who cannot be diagnosed as probable CAA based on criteria. However, these patients may have advanced CAA pathology, because CAA involvement propagates to deep areas in the later stage according to a pathologic study^12^. In this population, Aβ positivity may suggest advanced CAA pathology rather than hypertensive angiopathy. This is also supported by the finding of our previous study that Aβ + CAA patients had a greater burden of CAA MRI markers and a lower burden of hypertensive angiopathy MRI markers such as lacunes^7^. We consider delineating the probable underlying pathology important because it enables better prognostic prediction and appropriate treatment decisions^13,14^. Therefore, predicting Aβ positivity in patients with CAA MRI markers would be clinically useful, because it could help predict prognosis.

Among prediction models, machine learning methods have been getting much attention due to high predictive power and reliable performance. However, lack of the interpretability of the internal processing has become a major issue in machine learning research. To overcome this limitation, we chose two tree-based machine learning models: gradient boosting machine (GBM)^15^ and random forest(RF)^16^. These two methods can effectively quantify the relative importance of variables and provide their cut-off values, which provides clinically meaningful insights.

Therefore, we aimed to identify the most important variables (among imaging markers and clinical characteristics) and the optimal cut-off values of them (such as the number of lobar CMBs) to predict Aβ PET positivity using machine learning based models, in patients with suspected CAA MRI markers. We consider that this prediction model is going to help clinicians to easily select patients with poor prognosis, based on clinical and imaging findings only.

## Results

### Baseline characteristics

We recruited 71 participants, of whom 25 participants were Aβ- and remaining 46 participants were Aβ + . Mean Age (72.1 ± 7.5 vs. 75.0 ± 6.6, *p* = 0.098) and female ratio (15 vs. 22%, *p* = 0.327) were not different between the two groups. However, the Aβ- group showed a tendency of a higher prevalence of hypertension (40 vs. 18%, *p* = 0.050) and a higher rate of previous stroke (16 vs 9%, *p* = 0.045) compared with the Aβ + group. As a surrogate marker of CSVD, the number of lacunes was significantly higher in the Aβ- groups than in the Aβ + group (9.8 ± 13.1 vs 1.7 ± 2.5, *p* < 0.001). In terms of CAA markers, cSS was more commonly found in the Aβ + than in the Aβ- group (43.5 vs. 12%, *p* = 0.007). Number of lobar CMBs was also higher in the Aβ + group than the Aβ- group (26.3 ± 33.2 vs. 62.2 ± 80.4, *p* = 0.037). Although the number of superficial cerebellar CMBs was not different between the Aβ- and Aβ + groups (1.7 ± 4.4 vs. 1.8 ± 5.0, *p* = 0.994), the number of CMBs in cerebellar dentate nucleus was higher in the Aβ—group than in the Aβ + group (0.6 ± 1.0 vs. 0.2 ± 0.8, *p* = 0.049) (Table 1).

**Table 1:** Clinical characteristics of study participants.

	Total N = 71	Aβ ( −) N = 25	Aβ ( +) N = 46	*P* value
**Demographics**				
Age	74.0 ± 7.0	72.1 ± 7.5	75.0 ± 6.6	0.098
Female	37 (52.1)	15 (60.0)	22 (47.8)	0.327
Education years	9.9 ± 5.4	8.6 ± 5.4	10.6 ± 5.4	0.135
ApoE 4	28 (41.2)	6 (26.1)	22 (48.9)	0.071
ApoE 2	10 (14.7)	2 (8.7)	8 (17.8)	0.317
Hypertension	40 (56.3)	18 (72.0)	22 (47.8)	0.050
Diabetes	15 (21.1)	8 (32.0)	7 (15.2)	0.098
Dyslipidemia	20 (28.2)	10 (40.0)	10 (21.7)	0.102
Cardiac disease	5 (7.0)	0 (0.0)	5 (10.9)	0.087
Previous stroke	16 (22.5)	9 (36.0)	7 (15.2)	0.045
**Imaging markers**				
Number of lacunes	4.5 ± 8.8	9.8 ± 13.1	1.7 ± 2.5	< 0.001
Presence of cSS	23 (32.4)	3 (12.0)	20 (43.5)	0.007
Presence of lobar ICH	22 (31.0)	11 (44.0)	11 (23.9)	0.080
Number of lobar CMBs	49.5 ± 69.5	26.3 ± 33.2	62.2 ± 80.4	0.037
Number of deep CMBs	2.9 ± 6.4	4.7 ± 5.9	1.9 ± 6.5	0.075
Number of superficial cerebellar CMBs	1.8 ± 4.7	1.7 ± 4.4	1.8 ± 5.0	0.944
Number of CMBs in cerebellar dentate nucleus	0.3 ± 0.5	0.6 ± 1.0	0.2 ± 0.8	0.049

*Numbers are presented mean ± standard deviation or n (%).

Aβ = amyloid β**,** CMB = cerebral microbleeds, APO E = apolipoprotein E, cSS = cortical superficial siderosis, ICH = intracerebral hemorrhage.

### Important predictive variables for Aβ positivity

**Figure 1:**
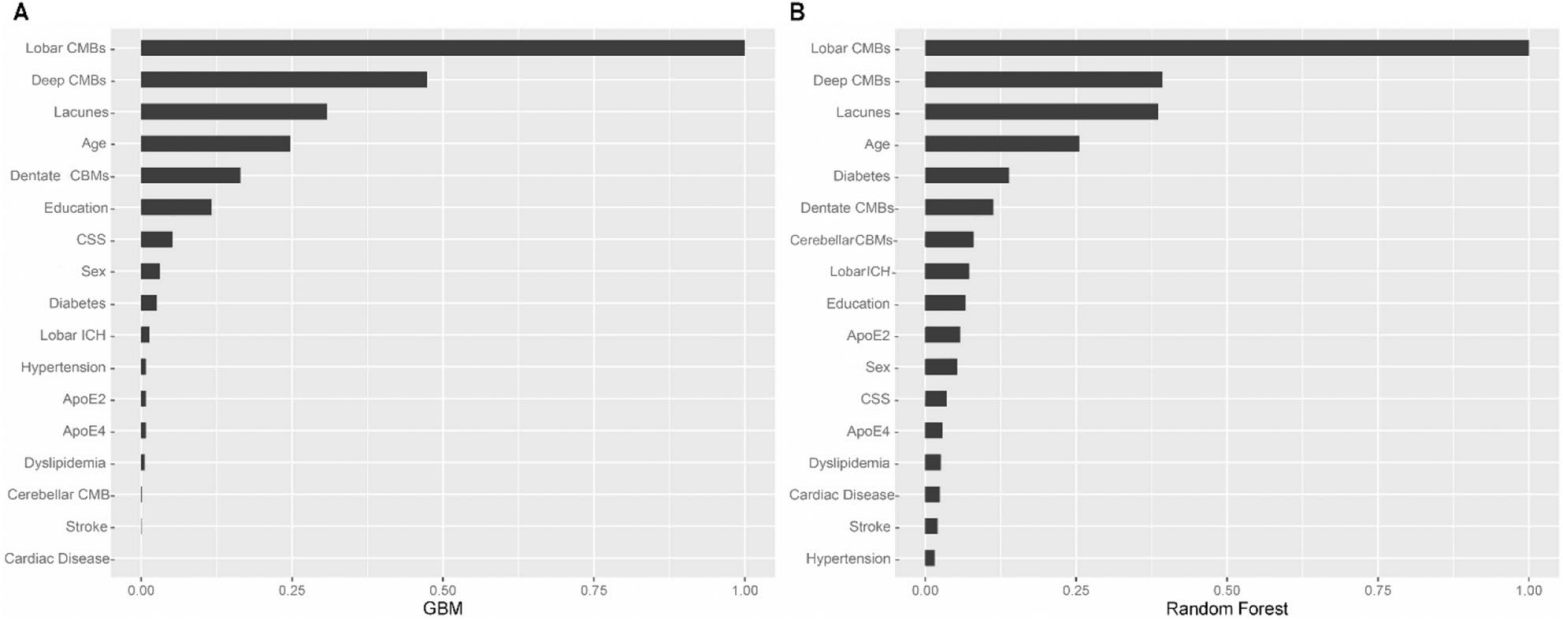
Importance plot of variables in GBM and RF models. GBM = gradient boosting model, RF = random forest, CMB = cerebral microbleed, cSS = cortical superficial siderosis, ICH = intracerebral hemorrhage, HTN = hypertension, APOE = apolipoprotein E. (**A**) Among 17 variables which were associated with CAA, the five important variables ranked in GBM model (relative importance) are as follows: the number of lobar CMBs (18.6), the number of deep CMBs (8.8), the number of lacunes (5.7), age (4.6), the number of CMBs in dentate nucleus. (3.1) (**B**) RF model chose the six important variables (relative importance) are as follows: the number of lobar CMBs (60.4), the number of deep CMBs (23.7), the number of lacunes (23.3), age (15.4), The absence of diabetes (8.4), and the number of CMBs in dentate nucleus (6.8).

Among 17 clinical and imaging variables, we computed relative importance using GBM and RF algorithms and selected the most important variables, which were similar in both models. The five important variables ranked in GBM model and their relative importance are as follows: the number of lobar CMBs (18.6), the number of deep CMBs (8.8), the number of lacunes (5.7), age (4.6), and the number of CMBs in dentate nucleus (3.1). On the other hand, RF model chose the six important variables as follows: the number of lobar CMBs (60.4), the number of deep CMBs (23.7), the number of lacunes (23.3), age (15.4), the absence of diabetes (8.4), and the number of CMBs in the dentate nucleus (6.8) (Fig. 1). After adding the lobar CMB/deep CMB ratio as a new variable, the highly ranked variables and their performance remained almost the same as the original result. (Supplementary Table S2).

### Cut-off values of predictive variables for Aβ positivity

**Figure 2:**
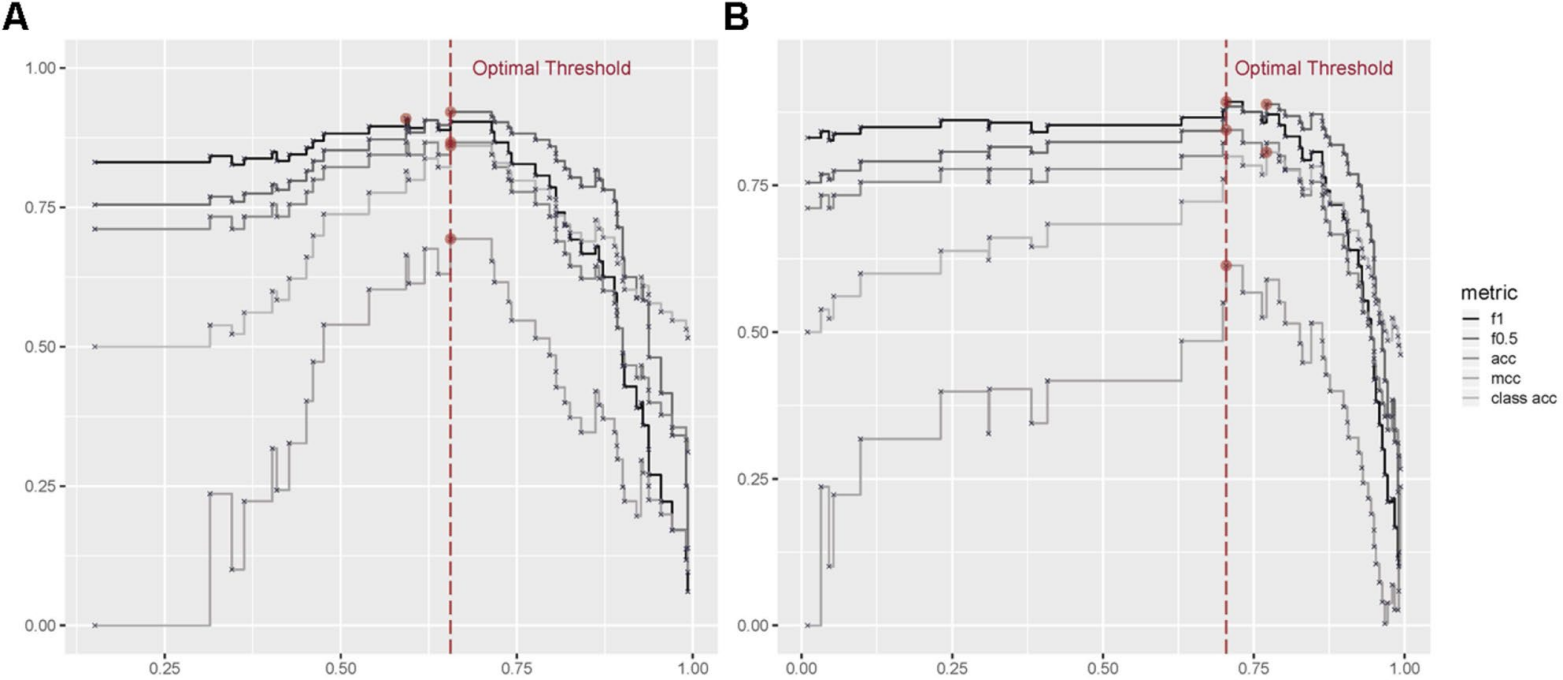
Detecting optimal threshold in multiple change points in GBM and RF models. GBM = gradient boosting model, ACC = accuracy, MCC = misclassification, Class ACC = class per accuracy, F1 = harmonic mean of the positive and negative predictive values with equal weights, F0.5 = mean of positive and negative predictive values, which gives more weight to PPV than to NPV. (**A**) In GBM method, the threshold was determined as 0.7043, when four metrics (F0.5, ACC, MCC, class ACC) are at their maximum values, respectively. (**B**) In RF method, threshold was determined as 0.6561, when three metrics (f1, ACC, MCC) are at their maximum values, respectively.

**Figure 3:**
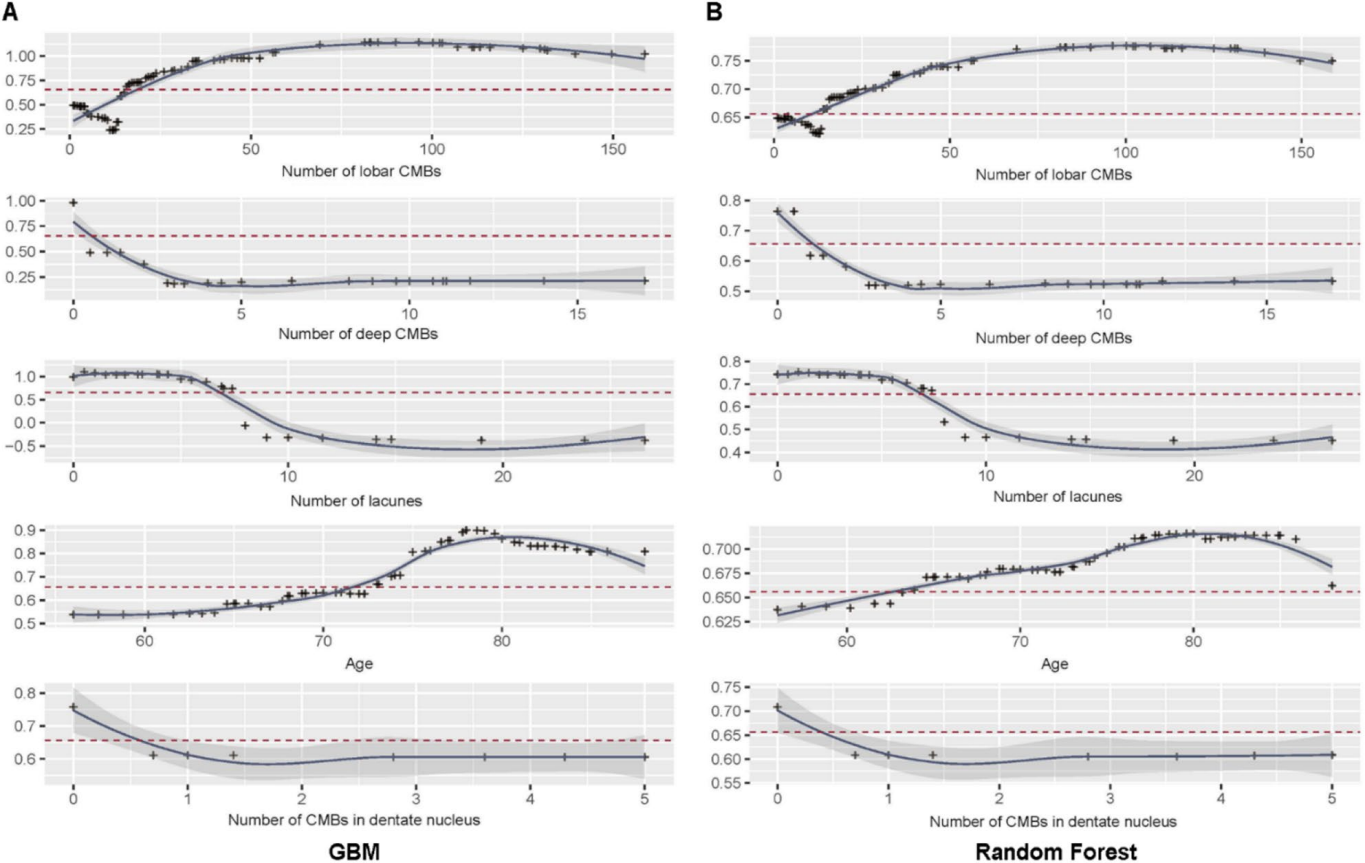
Cut-off values of important variables in GBM and RF models. GBM = gradient boosting machines, RF = random forest, CMBs = cerebral microbleeds, In PDP curve (y is threshold of metrics, and x is cut-off value), the optimal cut-off value was determined, when the curve passes the threshold which was obtained above. (**A**) Cut-off values of variables to predict Aβ positivity in GBM were as follows: (1) If the number of lobar CMB is more than 16.4, (2) if there is no deep CMBs, (3) if the number of lacunes is more than 7.4, (4) if age is older than 74.3 (GBM), (5) if there is no CMBs in dentate nucleus. (**B**) Cut-off values of variables to predict Aβ positivity in RF were as follows: (1) If the number of lobar CMB is more than 14.3 , (2) if there is no deep CMBs, (3) if the number of lacunes is more than 7.4, (4) if age is older than 64, (5) if there is no CMBs in dentate nucleus.

In GBM, the threshold was determined as 0.7043 when four metrics (F0.5, ACC, MCC, class ACC) were at their maximum values respectively. In RF, threshold was determined as 0.6561, when three metrics (F1, accuracy, misclassification) were at their maximum values, respectively.(Fig. 2) Using these thresholds obtained as above, we determined cut-off values of important variables. Cut-off values of variables to predict Aβ positivity were as follows: (1) If the number of lobar CMB is more than 16.4 (GBM)/14.3 (RF), (2) If there is no deep CMBs (GBM and RF), (3) If the number of lacunes is more than 7.4 (GBM and RF), (4) If age is older than 74.3 (GBM) /64(RF), (5) If there is no CMBs in dentate nucleus (Table 2, Fig. 3).

**Table 2:** Cut-off values of GBM and RF models.

	GBM	Random forest
Number of lobar CMBs	16.4	14.0
Number of deep CMBs	0	0
Number of lacunes	7.4	7.4
Age	74.3	63.9
Number of CMBs in dentate nucleus	0	0

GBM = gradient boosting model, CMB = cerebral microbleed.

### Model performances of GBM and RF based prediction models

Both GBM and RF models showed good performances; MSE was 0.14 ± 0.02 in GBM and 0.18 ± 0.06 in RF. RMSE was 0.41 ± 0.08 in GBM and 0.37 ± 0.03 in RF. Logarithmic loss was 0.47 ± 0.07 in GBM and 0.53 ± 0.17 in RF. Mean per class error was 0.22 ± 0.06 in GBM and 0.25 ± 0.14 in RF. Gini impurity was 0.65 ± 0.09 in GBM and 0.60 ± 0.24 in RF. AUC was 0.83 ± 0.04 in GBM and 0.80 ± 0.12 in RF. Precision-recall AUC was 0.86 ± 0.04 in GBM and 0.67 ± 0.18 in RF. (Table 3).

**Table 3:** The performance of GBM and RF models.

Performance measures (mean ± SD*)	GBM	Random forest
Mean square error	0.14 ± 0.02	0.18 ± 0.06
Root mean square error	0.37 ± 0.03	0.41 ± 0.08
Logarithmic loss	0.47 ± 0.07	0.53 ± 0.17
Mean per class error	0.22 ± 0.06	0.25 ± 0.14
Gini impurity	0.65 ± 0.09	0.60 ± 0.24
Area under curve	0.83 ± 0.04	0.80 ± 0.12
Precision-recall area under curve	0.86 ± 0.04	0.67 ± 0.18

*Lower values of mean square error, root mean square error, logarithmic loss, mean per class error, and gini impurity means better prediction power, and lower SD means higher reliability.

GBM = gradient boosting model; SD = standard deviation.

## Discussion

In present study, we developed machine-learning based models to predict Aβ positivity on PET in patients with suspected CAA markers. Our first major finding was that GBM and RF algorithms consistently ranked anatomical distribution of CMBs, age, the number of lacunes as the most important variables for predicting Aβ PET positivity. Our second major finding was the suggested cut-off values of these important variables (particularly, lobar CMBs higher than at least 14 and lacune number less than 7.4) predicting Aβ PET positivity. Finally, both models showed good performances, but GBM-based model performance was slightly better than RF-based model.

The first major finding was that both machine learning methods consistently ranked the number of lobar CMBs, deep CMBs, lacunes, and dentate nucleus CMBs, and age as the most important variables for prediction of Aβ PET positivity. Besides, the ranked orders of variables were similar in two models, although the value of relative importance was slightly different. Among selected variables, topographic location of CMBs (high number of lobar CMBs and absence of deep CMBs) and old age are well-known CAA predicting features according to the modified Boston Criteria. Therefore, it is reasonable that these factors could also predict Aβ PET positivity. However, the presence of cSS, which is considered as one of the important imaging parameters of CAA, was not highly ranked in our models. Considering that the prevalence of cSS was significantly higher in the Aβ + group than Aβ- group in our study, cSS seems to be associated with Aβ positivity, which is also consistent with previous study^17^. Nevertheless, cSS could be a less important predicative variable than topographic distribution of CMBs, number of lacunes, and age, when the model is made by the combination of various features in the memory clinic, which might be attributed to small number of patients having cSS in this clinical circumstance.

The second major findings were the cut-off values of important variables to predict Aβ positivity. First, both machine learning-based models showed that no CMB in deep structures and cerebellar dentate nucleus was predictive of Aβ positivity. We consider that our study finding supports the modified Boston criteria in which the presence of deep CMBs is exclusion criteria for probable CAA, even when number of lobar CMBs outweighs that of deep CMBs as in our cases; If these cases were advanced CAA as we hypothesized, presence of deep CMBs might not lower the possibility of Aβ positivity. This is along the same line with cerebellar dentate nucleus involvement. As equivalents of deep CMBs, CMBs in cerebellar dentate nucleus are likely due to hypertensive angiopathy as reported in recent studies^18,19^. Our prediction models suggested optimal cut-off values of lobar CMBs predicting Aβ positivity as 16.4 (GBM) or 14.3 (RF). Although, the modified Boston Criteria proposed that at least 2 lobar hemorrhages were enough to be diagnosed with probable CAA, some might argue that only two lobar CMBs could be found incidentally without CAA pathology. In addition, the previous study including patients with only CMBs reported that higher CMB counts increased specificity for predicting CAA^20^. This suggests the additional possibility that likelihood of CAA increases in a proportional relationship with CMBs number rather than a sharp threshold at ≥ 2 CMBs^20^. From this perspective, the cut-off of lobar CMBs (particularly in the absence of symptomatic lobar ICHs) for predicting Aβ positivity must be higher than two considering that Aβ positivity may relate to advanced CAA pathology. Therefore, machine learning methods derived cut-off values of lobar CMBs could be usefully applied to predict Aβ positivity, which is associated with CAA pathology and poor clinical prognosis in patients with only multiple CMBs even in the absence of symptomatic lobar ICHs.

Other noteworthy findings were the cut-off values of age and lacunes; The age cut-off for predict Aβ positivity were 74.3 (GBM) and 63.9 (RF), which were older than 55 years as presented in the modified Boston criteria. Although minimum age at CAA could develop is 55 years old according to the suggested criteria, this result shows that an older age increases the possibility of Aβ positivity in patients with CAA MRI markers. Finally, both prediction models showed that the number of lacunes lower than 7.4 was predictive of Aβ positivity. We consider that lacunes were considered as surrogate marker of hypertensive angiopathy rather than CAA. Therefore, when patients have mixed deep and lobar CMBs, the number of lacunes higher than 7.4 is almost always suggestive of hypertensive angiopathy, which is more likely to have negative Aβ PET scans. Nevertheless, the cut-off value of 7.4 was higher than expectation, which we considered was because FLAIR image with axial thickness of 2 mm (which is fivefold thinner than usual thickness of 10 mm) enabled sensitive counting of lacunes in study patients.

The final major finding was that both machine learning-based models showed good performance with higher than 80% of predictive accuracy, although GBM was slightly better than RF. We selected GBM and RF for the following reasons. First, previous large-scale studies have consistently suggested GBM and RF as robust ML algorithms^21–23^. Second, the generalizability may be ensured by comparing two methods with complementary methodological backgrounds. For example, although GBM performed better than RF on the skewed data, it could provide misleading outcomes from the noisy data and vice versa. Third, for more reliable predictions, we selected tree-based ML models and compared their interpretable predictions. Tree-based models provide the same interpretable methods such as relative importance and PDP. Variable importance determines the features that influence accurate classification^24^. Besides, PDP can estimate whether the variables had a positive or negative effect on the prediction using a marginal distribution. Thus, the intersection between the negative and positive PDPs provides cut-off values of the variables. In this study, GBM and RF showed similar interpretable results.

Especially, the importance of topographic distribution was reconfirmed by our machine learning methods. Particularly, new cut-off values of lobar CMBs and age in present study could be used as a supportive measure to predict Aβ positivity in patients with CAA MRI markers. Furthermore, the diagnosis of patients with many lobar CMBs combined with a few deep CMBs has been unclear. However, these models enable us to distinguish Aβ pathology from hypertensive angiopathy in this population by predicting Aβ positivity. We can also predict Aβ positivity using clinical information and MR imaging, which is less expensive and more readily available. Methodologically, the cut-off values have conventionally obtained, using receiver operating characteristic curve with only two metrics, sensitivity, and specificity. However, in this study, we obtained cut-off values using five metrics, which enabled higher dimensional analyses and consequently better accuracy than the conventional approach.

Our study has its strength in two machine learning based models (GBM and RF) which showed consistent and reliable results with good performances, although they independently select important variables and rank the important variables in supervised ways. We acknowledge some limitations of this study. There may exist concern about the overfitting problem in training models with a relatively small number of data samples. In addition, the cutoff values for variables such as age could have been biased due to the small sample size although we considered that the demographic data and the imaging features of this study population were reflective of the characteristics of patients with CAA markers who visited memory clinics. Therefore, future studies are required to develop more generalizable models with a possible external dataset. Also, we used Aβ positivity on PET instead of a pathologic confirmation. Nevertheless, prediction for Aβ positivity would be useful for clinicians to understand their clinical courses, based on clinical significance of Aβ PET positivity in CAA patients^7^. Finally, we used three different Aβ PET ligands in this study. However, this limitation may have been overcome, as previous studies demonstrated that three different PET uptakes are highly correlated with each other^25–27^.

In conclusion, we developed two reliable machine learning-based models to predict Aβ positivity in 71 patients with suspected CAA MRI markers using various clinical and imaging features, and they suggested useful clinical cut-offs for predictive variables. These models may help clinician to predict prognosis of patients with suspected CAA markers and to make stratified enrollment in clinical trials, by predicting Aβ PET positivity.

## Methods

### Participants

We included all 2333 patients who visited our memory clinic (Samsung Medical Center, Korea), complaining of cognitive impairment and underwent Aβ PET from September 2008 to June 2018. We scrutinized Brain MRI of all patients, and recruited patients who met the following criteria that we developed in this study: (1) If patients have at least one lobar ICH or cSS, only one lobar CMB is enough for them to be included; (2) If patients do not have either lobar ICH or cSS, 10 or more lobar CMBs are required for them to be included; (3) If patients have both lobar and deep CMBs, the number of lobar CMBs should be higher than that of deep CMBs. Therefore, we finally included 71 patients (26 PiB PET, 43 florbetaben PET, 2 flutemetamol PET) whom we refer to as “patients with suspected CAA markers” in this study.

We excluded patients with the presence of secondary causes of cognitive deficit (e.g. vitamin B12/folate, syphilis serology, and/or thyroid dysfunction), or structural lesion except for lobar ICH (e.g. territorial cerebral infarctions and brain tumors), or with psychiatric illness such as schizophrenia.

The Institutional Review Board of Samsung Medical Center approved the study protocol and informed consent was obtained from all subjects or, if subjects are under 18, from a parent and/or legal guardian.. This manuscript does not contain information or image that can lead to identification of a study participant. The methods were carried out in accordance with the approved guidelines.

### MR image acquisition

All participants underwent brain MRI including T2* GRE and fluid attenuated inversion recovery (FLAIR). The following parameters were used for the T2* GRE images: axial slice thickness, 5.0 mm; inter-slice thickness, 2 mm; repetition time (TR), 669 ms; echo time (TE) 16 ms; flip angle, 18°; matrix size, 560 × 560 pixels. The following parameters were used for the 3D FLAIR images: axial slice thickness of 2 mm; no gap; repetition time of 11 000 ms; echo time of 125 ms; flip angle of 90°; and matrix size of 512 × 512 pixels.

### Assessment of CMB, cSS, lobar ICH and lacunes on MRI

Imaging analysis was carried out by individuals who were trained in neuroimaging rating and blinded to the participant clinical details. All structural imaging markers of CSVD were rated in accordance with consensus guidelines^28,29^. Lobar CMBs were defined as homogenous and round lesions with signal loss (≤ 10 mm in diameter) on T2* GRE images, with location in exclusively lobar (cortex, gray-white matter junction, subcortical white matter) areas. Deep CMBs were defined as CMB in basal ganglia gray matter, internal and external capsules, and thalamus, according to brain observer microbleed scale (BOMBS)^30^.Infratentorial CMBs were also classified as deep CMBs. Cerebellar CMBs were separately counted and classified into dentate nucleus and superficial cerebellar CMBs^18^. cSS was defined as linear hypointensities on T2* GRE images consistent with chronic blood residues in the superficial layers of the cerebral cortex^31^. Lacunes were identified and counted in accordance with STRIVE (STandards for ReportIng Vascular changes on nEuroimaging)^28^.

### Aβ PET imaging acquisition

The mean value of MRI-Aβ PET interval was 8.8 ± 9.8 months. All patients underwent Aβ PET using a Discovery STe PET/CT scanner (GE Medical Systems, Milwaukee, WI) in a 3D scanning mode that examined 47 slices of 3.3 mm thickness spanning the entire brain. A 16-slice helical CT (140 keV, 80 mA; 3.75 mm section width) was performed for attenuation correction. For 11C-PiB PET, a 30-min emission static PET scan was performed 60 min after injection into an antecubital vein as a bolus of a mean dose of 420 MBq. For 18F-Florbetaben PET, a 20-min emission PET scan with dynamic mode (consisting of 4 × 5 min frames) was performed 90 min after injection into an antecubital vein as a bolus of a mean dose of 381 MBq. For flutemetamol, 20-min emission static PET scan with dynamic mode (consisting of 4 × 5 min frames) was performed 90-min after injection into an antecubital vein as a bolus of a mean dose of 185 MBq.

### Aβ PET image preprocessing and definition for Aβ positivity

Both MR and Aβ PET images were co-registered with each other using the rigid-body transformation. The T1-weighted MR image of each subject was aligned with the MNI-152 template using a non-linear deformation including translation, rotation, scaling and shearing. After standard space registration, we divided grey matter into 116 regions using the Automated Anatomical Labeling atlas^32^. In order to compute standardized uptake value ratios (SUVR) for PiB and florbetaben^33^, every voxel intensity was normalized by the mean intensity of cerebellum regions. For flutemetamol PET, we computed SUVR by the mean intensity of pons regions as reference value. We defined Aβ positivity on each PET as follows: (1) If global PiB SUVR (assessed from the volume-weighted average SUVR of 28 bilateral cerebral cortical VOIs) was greater than 1.5, (2) If visual rating score on florbetaben PET was 2 or 3 on the brain Aβ plaque load (BAPL) scoring system^34^, or (3) If any one of the brain regions systematically reviewed for ^18^F-flutemetamol PET was positive in either hemisphere^34^.

### Statistical analysis

We compared the demographic and clinical characteristics between the Aβ+ and Aβ− groups using Student t-tests for continuous variables and chi-square test for dichotomous variables. Statistical analyses were performed using R version 3.5.0.

### Potential variables for predicting Aβ positivity

We included all clinical and imaging characteristics as potential variables in model development: gender, education year, vascular risk factors (dyslipidemia, diabetes, cardiac disease, previous stroke, and hypertension), apolipoprotein E (APOE) genotype, number of CMBs in each location (number of lobar CMBs, deep CMBs, dentate nucleus CMBs, and superficial cerebellar CMBs), presence of lobar ICH and cSS, and number of lacunes.

For sensitivity analysis, we performed the same analysis with the lobar CMB/deep CMB ratio as an additional variable. If the number of deep CMBs was zero, we used the number of lobar CMBs instead of the lobar CMB/deep CMB ratio.

### Model generation for classifying Aβ positivity

Among the tree-based ML models, we selected GBM and RF. GBM generates accurate classifiers using linear combinations of the base classifiers adjusted by their weights iteratively. The PDP approach was originally introduced by J.H. Friedman in the GBM paper^15^. RF creates multiple decision trees using bootstrap samples and the binning of outliers. RF aggregates their decisions by averaging or majority voting^35^. GBM and RF analysis were carried out using different combinations of hyperparameter settings and varying search criteria in randomly selected trials. In grid search process, the advanced computing power enabled searching the entire hyperparameter space.

Twenty repetitions of tenfold cross-validation (CV) were conducted in order to select the optimal solution^36^. K-fold CV is to divide the data set into non-overlapping k equal partitions. Each data partition is then used as the validation set and the remaining K-1 partitions are used as a training set. We selected K = 10 as an empirically ideal situation of 10 training sets and 10 validation sets^37^. Under the CV procedure, the generalization of predictive power and validation errors were computed. The best parameter setting corresponding to the minimal error obtained by CV, was then applied to train the model using a train set and a validation set, which were 70% and 15% of the entire data set respectively. Remaining 15% data set was used as a test set, and their performance was estimated. The whole process was repeated over 20 times in order to evaluate reliable classifier performances.

Although we selected GBM and RF in this study, we compared the performance of other ML methods such as logistic regression^38 ^,k-nearest neighbors (KNN)^39^, and support vector machine (SVM)^40^. Further details on the classifiers are provided in Supplementary Method 1. For a fair comparison, the same CV data partitions were used across all the ML models, and performance was estimated using the arithmetic means of the outcome. Supplementary Table S1 and Supplementary Method 2 provide the details on model performance and additional performance measures, respectively.

### Interpretable machine learning

For each analysis, the extent to which the variables influenced the accuracy of classification was quantified by calculating the relative variable importance^41^. In the tree-based model such as GBM and RF, when the variable split the tree, relative importance value of that variable was estimated by discrepancy of the squared error loss over all tree. A higher relative importance value indicates greater influence of the variable in classifying Aβ positivity.

Optimal threshold was estimated in terms of F1 score, F0.5 score, accuracy, misclassification and class accuracy which are widely recommended for classification tasks^42^.

The cut-off values of the important, numerical variables were determined by partial dependence plot (PDP) which is a graphical representation tool describing the relationship between target feature and input features resulting by importance variables. Let $\mathbf{x}$ be the space of input variables consisting of a chosen subset space and its complemental space,

$${{x}}_{{s}}\cup {{x}}_{{c}}=\mathbf{x}$$

Then the approximation $\widehat{{F}}(\mathrm{x})$ depend on both subset space.

$$\widehat{\varvec{F}}(\text{x})= \widehat{\varvec{F}}({\varvec{x}}_{\varvec{s }},{\varvec{x}}_{\varvec{c}}), {\widehat{\varvec{F}}}_{\varvec{c}}\left({\varvec{x}}_{\varvec{s}}\right)= \widehat{\varvec{F}}\left({\varvec{x}}_{\varvec{s }}|{\varvec{x}}_{\varvec{c}}\right)$$

$${\overline{\varvec{F}}}_{\varvec{s}}\left({\varvec{x}}_{\varvec{s}}\right)= {\varvec{E}}_{{\mathbf{x}}_{\varvec{c}}}\left[\widehat{\varvec{F}}(\text{x})\right]=\int \widehat{\varvec{F}} \left({\varvec{x}}_{\varvec{s }},{\varvec{x}}_{\varvec{c}}\right){\varvec{p}}_{\varvec{c}}\left({\varvec{x}}_{\varvec{c}}\right){\varvec{dx}}_{\varvec{c}}$$

$${\widetilde{\varvec{F}}}_{\varvec{s}}\left({\varvec{x}}_{\varvec{s}}\right)= {\varvec{E}}_{\mathbf{x}}[\widehat{\varvec{F}}(\mathbf{x})|{\varvec{x}}_{\varvec{s}}]=\int \widehat{\varvec{F}}(\mathbf{x}){\varvec{p}}_{\varvec{z}}({\varvec{x}}_{\varvec{c}}|{\varvec{x}}_{\varvec{s}}){\varvec{dx}}_{\varvec{c}}$$

In PDP curve (y is threshold of metrics, and x is cut-off value), the optimal cut-off value was determined, when the curve passes the threshold which was obtained above.

### Assessment of model performance

To assess model performance of prediction model, we used six measures as follows: mean square error (MSE), root mean square error (RMSE), logarithmic loss, mean per class error, area under curve, precision-recall area under curve (AUC), gini impurity. We computed the mean values of each measure after 20 iterations.

The MSE of estimator (of a procedure for estimating an unobserved quantity) measures the average of the square of the error- that is, the average squared difference between the estimated values and the actual value. The less MSE means better prediction. The tracking task was scored by calculating the RMSE between the target and response signals^43^. Logarithmic loss (related to cross-entropy) increases as the predicted probability diverges from the actual label. Mean Per Class Error is the average of the errors of each class in multi-class data set, which measures misclassification of the data across the classes. AUC is used to evaluate how well a binary classification model can distinguish true positives from false positives. Precision-Recall curves summarize the trade-off between the true positive rate and the positive predictive value for a predictive model using different probability thresholds especially for imbalanced dataset. Gini impurity is a measure of how often a randomly chosen element from the set would be incorrectly labeled if it was randomly labeled according to the distribution of labels in the subset.

## Supplementary information

Supplementary Information

## Data Availability

The data sets generated or analyzed during the current study are available from the corresponding author upon reasonable request.
