# Supplementary material for: Prediction of amyloid β PET positivity using machine learning in patients with suspected cerebral amyloid angiopathy markers
Source: Sci Rep. 2020 Nov 2;10:18806. doi: 10.1038/s41598-020-75664-8 (PMC7608617; doi:10.1038/s41598-020-75664-8)
Supplement: Supplementary file 1 — Supplementary Information [file 41598_2020_75664_MOESM1_ESM.docx]

**Prediction of amyloid** β **PET positivity using machine learning in patients with suspected cerebral amyloid angiopathy markers**

**Young Hee Jung, MD^1,2,3^ꭞ, Hyejoo Lee PhD^2,3,4^ꭞ, Hee Jin Kim, MD, PhD^2,3,4^, Duk L. Na, MD, PhD^2,3,4,6,7^, Hyun Jeong Han, MD, PhD^1^**, **Hyemin Jang, MD, PhD^2,3,4*^, Sang Won Seo, MD, PhD^2,3,4,5*^**

ꭞ * All authors listed contributed equally to this study.

^1^Department of Neurology, Myoungji Hospital, College of Medicine, Hanyang University, Goyang, Republic of Korea

^2^Department of Neurology, Samsung Medical Center, Sungkyunkwan University School of Medicine, Seoul, Republic of Korea

^3^Neuroscience Center, Samsung Medical Center, Seoul, Republic of Korea

^4^Samsung Alzheimer Research Center, Research Institute for Future Medicine, Samsung Medical Center, Seoul, Republic of Korea

^5^Department of Clinical Research Design & Evaluation, SAIHST, Sungkyunkwan University, Seoul, Republic of Korea

^6^Department of Health Science and Technology, SAIHST, Sungkyunkwan University, Seoul, Republic of Korea

^7^Stem Cell & Regenerative Medicine Institute, Samsung Medical Center, Seoul, Republic of Korea

**Supplementary Information**

**Supplementary Method 1.** Methodological background of k-nearest neighbor (KNN) and support vector machine (SVM)

**Supplementary Method 2.** Additional model performance measures

**Supplementary Table S1.** Additional model performance

**Supplementary Table S2.** Model performance including variable for lobar CMB/deep CMB ratio

**Supplementary Table S3.** Variable importance of GBM and RF including variable for lobar CMB/deep CMB ratio

**Supplementary Table S4.** Comparison of model performance between logistic regression and other ML methods including variable for lobar CMB/deep CMB ratio

This supplementary material has been provided by the authors to give readers additional information about their work.

**Supplementary Method 1.** Methodological information of k-nearest neighbor (KNN) and support vector machine (SVM)

In classification, the k-nearest neighbor (KNN) algorithm is one of the fundamental and simplest nonparametric classifies by finding k number of nearest neighbors from the training dataset. The nearest neighbor is estimated by Euclidean distance and the number of k is user defined positive integer, typically a small constant. The classification decision is made by taking majority voting of its neighbors, which can be biased on the skewed data set.

The standard SVM is a binary classifier finding a hyperplane which divides the data space with a maximum margin (the distance between the hyperplane and the nearest point). The hyperplane is constructed by these data points which are known as support vectors. However, there might not exist a hyperplane that can separate all data points. In that case, SVM can use a soft margin that minimizes training error. If the data are not linearly separable in the original feature space, they are transformed by applying the kernel trick to a higher dimensional space, where the data become linearly separable. There are commonly used kernels in SVM such as linear, polynomial, Gaussian radial basis function and hyperbolic tangent.

**Supplementary Method 2.** Additional model performance measures

The performance of these models was evaluated by area under the ROC curve (AUROC), overall accuracy (ACC), sensitivity, specificity, positive predictive value (PPV) and negative predictive value (NPV), F1, balanced accuracy.

Overall accuracy was obtained by the total number of correct predictions divided by the total number of predictions. Sensitivity measures the proportion of positive predictions among actual positives and specificity is the proportion of negative predictions among actual negatives. PPV is the proportion of true positives among the positive predictions and NPV is the proportion of true negatives among the negative predictions. F1 score is the harmonic mean of precision and recall score and balanced accuracy is the mean of sensitivity and specificity.

**Supplementary Table S1.** Additional model performance

|  | **AUROC** | **Accuracy** | **Sensitivity** | **Specificity** | **PPV** | **NPV** | **F1** | **Balanced Accuracy** |
| --- | --- | --- | --- | --- | --- | --- | --- | --- |
| Logistic regression | 0.72 | 0.70 | 0.41 | 0.87 | 0.62 | 0.73 | 0.49 | 0.637 |
| RF | 0.80 | 0.78 | 0.55 | 0.91 | 0.76 | 0.79 | 0.64 | 0.727 |
| GBM | 0.83 | 0.76 | 0.84 | 0.60 | 0.80 | 0.68 | 0.82 | 0.722 |
| KNN | 0.77 | 0.73 | 0.57 | 0.82 | 0.63 | 0.78 | 0.61 | 0.696 |
| SVM | 0.83 | 0.77 | 0.90 | 0.54 | 0.78 | 0.75 | 0.84 | 0.722 |

AUROC=area under the ROC curve; PPV=positive predictive value; NPV=negative predictive value; F1= harmonic mean of PPV and NPV; RF=random forest; GBM=gradient boost machine; KNN=k-nearest neighbors; SVM= support vector machine

**Supplementary Table S2**. Model performance including variable for lobar CMB/deep CMB ratio

|  | AUROC | Accuracy | Sensitivity | Specificity | PPV | NPV | F1 | Balanced Accuracy |
| --- | --- | --- | --- | --- | --- | --- | --- | --- |
| Logistic regression | 0.73 | 0.72 | 0.85 | 0.49 | 0.75 | 0.64 | 0.80 | 0.67 |
| RF | 0.80 | 0.74 | 0.84 | 0.56 | 0.78 | 0.65 | 0.81 | 0.70 |
| GBM | 0.83 | 0.76 | 0.84 | 0.60 | 0.80 | 0.68 | 0.82 | 0.72 |
| KNN | 0.78 | 0.75 | 0.86 | 0.53 | 0.77 | 0.68 | 0.82 | 0.70 |
| SVM | 0.84 | 0.75 | 0.84 | 0.60 | 0.79 | 0.67 | 0.81 | 0.72 |

AUROC=area under the ROC curve; PPV=positive predictive value; NPV=negative predictive value; F1= harmonic mean of PPV and NPV; RF=random forest; GBM=gradient boost machine; KNN=k-nearest neighbors; SVM= support vector machine

**Supplementary Table S3.** Variable importance of GBM and RF including variable for lobar CMB/deep CMB ratio

| **GBM** | |  |  | **RF** | |
| --- | --- | --- | --- | --- | --- |
| Variable | Importance |  |  | Variable | Importance |
| Number of lobar CMB | 11.2 |  |  | Lobar CMB/ deep CMB ratio | 5.6 |
| Lobar CMB/deep CMB ratio | 6.6 |  |  | Number of lobar CMBs | 5.3 |
| Number of deep CMBs | 5.1 |  |  | Number of_deep CMBs | 5.0 |
| Number of Lacune | 4.8 |  |  | Number of Lacune | 3.8 |
| cSS | 4.1 |  |  | Age | 2.1 |
| Age | 3.3 |  |  | cSS | 2.1 |
| Education | 2.9 |  |  | Education | 1.5 |
| Number of dentate CMB | 1.8 |  |  | ICH | 1.4 |
| ICH | 0.9 |  |  | ApoE4 | 1.0 |
| HTN | 0.8 |  |  | Gender | 1.0 |
| ApoE4 | 0.6 |  |  | number of_dentate CMBs | 0.6 |
| Diabetes | 0.5 |  |  | Diabetes | 0.3 |
| ApoE2 | 0 |  |  | ApoE2 | 0 |
| Cardiac disease | 0 |  |  | Cardiac disease | 0 |
| DM | 0 |  |  | HTN | 0 |
| Dyslipidemia | 0 |  |  | Dyslipidemia | 0 |
| Number of Superficial cerebellar CMB | 0 |  |  | Number of Superficial cerebellar CMB | 0 |
| Stroke | 0 |  |  | Stroke | 0 |

CMB = cerebral microbleeds, APOE = apolipoprotein E, cSS = cortical superficial siderosis, ICH = intracerebral hemorrhage

**Supplementary Table S4.** Comparison of model performance between logistic regression and other ML methods including variable for lobar CMB/deep CMB ratio

|  | **AUROC** | ***p*-value** |
| --- | --- | --- |
| Logistic regression | 0.73 | [reference] |
| RF | 0.80 | 0.000148 |
| GBM | 0.83 | < 0.0001 |
| KNN | 0.78 | < 0.0001 |
| SVM | 0.84 | < 0.0001 |

*p*-values for the DeLong test comparing area under the receiver operating characteristic curves for different models with logistic regression.

AUROC=area under the ROC curve; RF=random forest; GBM=gradient boost machine; KNN=k-nearest neighbors; SVM= support vector machine
